# Pretreatment of Epithelial Cells with Live *Streptococcus pneumoniae* Has No Detectable Effect on Influenza A Virus Replication *In Vitro*

**DOI:** 10.1371/journal.pone.0090066

**Published:** 2014-03-03

**Authors:** Kang Ouyang, Shireen A. Woodiga, Varun Dwivedi, Carolyn M. Buckwalter, Anirudh K. Singh, Basavaraj Binjawadagi, Jagadish Hiremath, Cordelia Manickam, Rose Schleappi, Mahesh Khatri, Jianmin Wu, Samantha J. King, Gourapura J. Renukaradhya

**Affiliations:** 1 College of Animal Science and Technology, Guangxi University, Nanning, China; 2 Food Animal Health Research Program (FAHRP), OARDC, Department of Veterinary Preventive Medicine, The Ohio State University, Wooster, Ohio, United States of America; 3 Center for Microbial Pathogenesis, Nationwide Children's Hospital, Columbus, Ohio, United States of America; 4 Department of Pediatrics, The Ohio State University, Columbus, Ohio, United States of America; Virginia Polytechnic Institute and State University, United States of America

## Abstract

Influenza A virus (IAV) and *Streptococcus pneumoniae* (pneumococcus) are two major upper respiratory tract pathogens responsible for exacerbated disease in coinfected individuals. Despite several studies showing increased susceptibility to secondary bacterial infections following IAV infection, information on the direct effect of *S. pneumoniae* on IAV *in vitro* is unknown. This is an important area of investigation as *S. pneumoniae* is a common commensal of the human upper respiratory tract, present as an important coinfecting pathogen with IAV infection. A recent study showed that *S. pneumoniae* enhances human metapneumovirus infection in polarized bronchial epithelial cells *in vitro*. The aim of the current study was to determine whether treatment of epithelial cells with *S. pneumoniae* affects IAV replication using a standard immunofluorescence assay (IFA). For this study we used four IAV permissive epithelial cell lines including two human-derived cell lines, 12 pneumococcal strains including recent human clinical isolates which represent different genetic backgrounds and serotypes, and six IAV strains of varying genetic nature and pathogenic potential including the pandemic 2009 H1N1 virus. Our results suggested that pretreatment of MDCK cells with 7.5×10^6^ colony-forming units (CFUs) of live *S. pneumoniae* resulted in gradual cell-death in a time-dependent manner (0.5 to 4 hr). But, pretreatment of cell lines with 7.5×10^5^ and lower CFUs of *S. pneumoniae* had no detectable effect on either the morphology of cells or on the IAV replication. However, unlike in epithelial cell lines, due to influence of secreted host factors the effect of pneumococci on IAV replication may be different during coinfections *in vivo* in the human upper respiratory tract, and *in vitro* with primary human polarized bronchial epithelial cells.

## Introduction

Influenza A virus (IAV) causes greater than 250,000 deaths annually in the industrialized world [1], and bacterial infections frequently cause secondary illnesses during influenza outbreaks [2], [3]. The IAV pandemics of the 20^th^ century (1918, 1957, and 1968) clearly demonstrated that infection with IAV facilitates the progression of *S. pneumoniae* from a commensal organism to a potentially fatal pathogen [2], [4]. Historically, most research on infectious diseases has focused on infections with single pathogens. However, infections with pathogens often occur in the context of preexisting viral and bacterial infections [5]. Despite several studies showing increased susceptibility to secondary bacterial infection following IAV infection [6], [7], other studies have shown that pretreatment of *S. pneumoniae* or its lysates led to induction of interferons, cytokines and chemokines which mitigate disease severity of IAV infection [5]. Although *S. pneumoniae* is an important human pathogen, it is also a common commensal of the human respiratory tract which colonizes approximately 50 to 70% of children aged 2–3 years, and also in approximately 10% of adults [8]. The synergistic effect of coinfection with *S. pneumoniae* and IAV has been studied *in vivo* using mouse models which revealed the interaction of the organisms, the host immune status and its activation in the host [9], [10]. However, due to lack of colonization of all the pathogenic strains of *S. pneumoniae* and infection of IAV strains in rodent models, in this study *in vitro* analysis was chosen instead of *in vivo*. Furthermore, a recent study demonstrated that *S. pneumoniae* enhances human metapneumovirus infection in polarized bronchial epithelial cells [11]. Our hypothesis was that *S. pneumoniae* increases influenza viral replication, thereby contributing to severity of disease. The aim of the current study was to determine whether pretreatment of epithelial cells with *S. pneumoniae* affects IAV infection in different IAV permissive cell types.

## Materials and Methods

### Cell propagation

Four epithelial cell types, Madin-Darby canine kidney cell line (MDCK) [12], porcine lung respiratory epithelial cell line (MK1-OSU) (this cell line was developed from tracheobronchial tissues of a pig, gifted by Dr. Mahesh Khatri, FAHRP, OARDC, Wooster, unpublished), human lung adenocarcinoma epithelial cell line (A549, ATCC CCL-185) [13], and human pharyngeal carcinoma cell line (D562, ATCC CCL-138) [14] were used in this study. All four cell lines were maintained as described previously [15], [16]. Briefly, cells were grown in Dulbecco's Modified Eagle Medium (DMEM) (Hyclone, MA) supplemented with 10% fetal bovine serum (Sigma, MO), 0.1 mM HEPES (Sigma, MO), and antibiotic-antimycotic (Gibco BRL, NY) mixture at 37°C in a humidified atmosphere with 5% CO2. Cell monolayers were detached by using trypsin (final concentration, 0.25%) and EDTA (final concentration, 0.02%) (Gibco BRL, NY) and seeded at a cell density of 2×10^4^ viable cells per well of a 96-well tissue culture plate with a low evaporation lid (BD Falcon, NJ). Plates were used following overnight incubation when greater than 90% confluence was observed.

### Bacterial strains and growth conditions

Twelve pneumococcal strains were selected to represent different genetic backgrounds and serotypes (Table 1). *S. pneumoniae* were grown in Todd Hewitt broth containing 0.2% yeast extract (both from Becton Dickinson, CA) (THY). All *S. pneumoniae* strains were stored at -80°C and the bacterial cells were picked using a sterile swab and patched on a 5% sheep's blood agar plate and incubated in 5% CO2 for 16 hr. A single colony was picked into 5 mL of THY medium and grown to mid exponential phase (OD_600_ = 0.25 to 0.6), glycerol was added to a 15% v/v ratio and then fast frozen and stored at −80°C as a starter culture. *S. pneumoniae* starter cultures were thawed and used to inoculate 5 mL of THY medium, and they were grown at 37°C to mid log phase and four samples OD (0.2 to 0.6 OD600) taken were used for plotting calibration curves for each strain. The exact CFUs were enumerated by a serial dilution plating method. Bacterial CFUs used in the following experiments were determined based on their respective calibration curve.

**Table 1:** Representative data showing the effect of pneumococcal (TIGR4) product (supernatant) on IAV (A/swine/Ohio/24366/07) replication in MDCK cells.

Dilution of the sup/medium	Duration of treatment	Treat with TIGR4 sup in growth medium	Treat with only TIGR4 growth medium	Only pretreat and IAV infection	Both pre- and post-treat and IAV infection	IAV titer TCID_50_ per ml
1:1	0.5 hr	+	-	+	-	3.2×10^4^
1:1	0.5 hr	+	-	-	+	3.2×10^5^
1:1	0.5 hr	-	+	+	-	3.2×10^4^
1:1	0.5 hr	-	+	-	+	3.2×10^5^
1:10	0.5 hr	+	-	+	-	3.2×10^4^
1:10	0.5 hr	+	-	-	+	3.2×10^5^
1:10	0.5 hr	-	+	+	-	3.2×10^4^
1:10	0.5 hr	-	+	-	+	3.2×10^5^
1:1	6 hr	+	-	+	-	3.2×10^4^
1:1	6 hr	+	-	-	+	3.2×10^5^
1:1	6 hr	-	+	+	-	5.6×10^4^
1:1	6 hr	-	+	-	+	3.2×10^5^
1:10	6 hr	+	-	+	-	3.2×10^4^
1:10	6 hr	+	-	-	+	3.2×10^5^
1:10	6 hr	-	+	+	-	3.2×10^4^
1:10	6 hr	-	+	-	+	3.2×10^5^
1:1	12 hr	+	-	+	-	3.2×10^4^
1:1	12 hr	+	-	-	+	3.2×10^5^
1:1	12 hr	-	+	+	-	3.2×10^4^
1:1	12 hr	-	+	-	+	3.2×10^5^
1:10	12 hr	+	-	+	-	3.2×10^4^
1:10	12 hr	+	-	-	+	3.2×10^5^
1:10	12 hr	-	+	+	-	5.6×10^4^
1:10	12 hr	-	+	-	+	3.2×10^5^
1:1	24 hr	+	-	+	-	3.2×10^4^
1:1	24 hr	+	-	-	+	3.2×10^5^
1:1	24 hr	-	+	+	-	5.6×10^4^
1:1	24 hr	-	+	-	+	5.6×10^5^
1:10	24 hr	+	-	+	-	3.2×10^4^
1:10	24 hr	+	-	-	+	1.8×10^5^
1:10	24 hr	-	+	+	-	5.6×10^4^
1:10	24 hr	-	+	-	+	3.2×10^5^
-	-	-	+	-	-	5.6×10^4^
-	-	-	-	-	-	3.2×10^5^

Cells were treated (pre and/or post) with *S. pneumoniae* (TIGR4) culture supernatant (sup) at indicated dilutions and time, and infected with A/swine/Ohio/24366/07 at MOI 0.1. Cell culture supernatants harvested at 24 hr post-infection were analyzed to determine the viral titers using MDCK cells by the IFA method.

### Ethics Statement

This study was carried out in strict accordance with the institutional biosafety committee recommendations, The Ohio State University (Protocol Number: 2012R00000006) and Nationwide Children's Hospital (Protocol Number: IBS00000046). The collection of tissues from pigs to make the cell line used in the study was carried out in strict accordance with the recommendations by Public Health Service Policy, United States Department of Agriculture Regulations and the Federation of Animal Science Societies' Guide for the Care and Use of Agricultural Animals in Agricultural Research and Teaching; and all relevant institutional, state, and federal regulations and policies regarding animal care and use at The Ohio State University. The protocol to collect tissue samples from the pig respiratory tract to use in immune response study and for growing cells in culture to make cell lines was approved by the Institutional Animal Care and Use Committee of The Ohio State University (Protocol Numbers: 2008-AG028). All the pigs were maintained, samples collected, and euthanized, and necessary efforts were made to minimize suffering.

### Virus propagation

Six IAV strains of swine and human origin were used in the study, pandemic H1N1 (A/California/04/2009), MN01 swH1N2, and H3N2 virus (A/Sw/Texas/1998) were provided by Dr. Sagar Goyal (Veterinary Population Health, University of Minnesota); H3N2 virus (Sw/CO/99) was provided by Dr. Richt (Department of Diagnostic Medicine and Pathobiology, Kansas State University); Swine H1N1 viruses, A/swine/Ohio/24366/07 [17] and A/swine/Ohio/75004/04 [17] were provided by Dr. Mo Saif (FAHRP, OARDC, OSU). Virus stocks were prepared in MDCK cells as previously described [18]. Briefly, IAV were inoculated into MDCK cells in serum free DMEM with tocylsulfonyl phenylalanyl chloromethyl ketone (TPCK)-trypsin (Sigma, MO) (1 µg/mL), and 2–3 days after infection virus-containing supernatants were collected and stored at −70°C.

### Virus Titration

Titers of viral stocks were determined by indirect immunofluorescence assay (IFA) using MDCK cells. Ten-fold serially diluted virus samples with TPCK-trypsin (1 µg/mL) were added to MDCK cell monolayers grown in a 96-well tissue culture plate. After 24 hr of incubation, IFA was performed as described previously [18]. Briefly, cell monolayers were washed once in PBS, fixed with 100 µL/well of 80% acetone in milli Q water for 10 min, liquid discarded, plates dried in the fume hood for approximately 30 min, and finally the cells were soaked in PBS-0.05% Tween 20 (PBS-Tween) for 5 min. Cells were subsequently incubated with IAV nucleoprotein specific monoclonal antibody (Cal Bioreagents, M058) (1∶5,000) at 37°C for 2 hr. After washing with PBS-Tween, Alexa Fluor 488 conjugated goat anti-mouse IgG (H+L) secondary antibody (Invitrogen, A11029) (1∶4,000) was added and incubated for 1.5 hr. Stained cells were washed with PBS-Tween and preserved with a mounting medium (60% glycerol in PBS). Cells were examined for the presence of fluorescent-staining cells using an Olympus IX51 microscope with a FITC wide pass filter set. The viral titer was calculated using the Reed and Muench method and expressed as TCID_50_ per mL as described previously [18], [19]. The number of fluorescent focal units (FFU) per mL was then calculated as TCID_50_ per mL [20] (Table 1).

### Quantification of IAV and *S. pneumoniae* for pretreatment and infection study

All four epithelial cell lines were infected with a range of multiplicities of infection (MOI) 1, 0.1, 0.01, and 0.001 to determine the required amount of IAV showing approximately 100 fluorescent focal units (FFU) per well at post-20 hr infection. We performed this study as infectivity of the six strains of IAV in the four epithelial cell lines was not identical. This initial study has helped us to select the ideal virus quantity which practically enabled us to count FFU in the range of 50 -150 in each well of the 96-well plate, a number which would allow us to determine the impact of pretreatment with 12 different pneumococcal strains on IAV replication.

Similarly, to determine the appropriate bacterial CFUs for pretreatment of the four cell types without affecting cell viability, we performed a pneumococcal inoculum dependent cell viability and IAV infection experiment. For this standardization assay we selected, one cell line (MDCK cells), *S. pneumoniae* strain (TIGR4), and IAV strain (SwH1N1/OH/2007/43266). MDCK cells were grown to 90% confluence in a 96-well plate as described above, washed with PBS prior to incubation with different CFUs of live TIGR4 cells (7.5×10^2^ to 7.5×10^6^ per well) in triplicate wells. Cells treated with THY medium were included as a control. After each time point of bacterial incubation (0.5, 1, 2, and 4 hr) the designated wells were washed three times with PBS to remove the seeded bacteria. Subsequently, cells were infected with an IAV at 0.01 MOI in DMEM containing antibiotics or treated with the infection medium as a control for 20 hr. After the initial viral adsorption period of 1 hr, cells were washed with PBS and serum free DMEM was added to all the wells. An IFA was performed as described above to enumerate virus infected cells at 20 hr post-infection. This experiment was repeated twice and the results were consistent between experiments.

### Effect of *S. pneumoniae* products on IAV replication

MDCK cell monolayers grown in 96-well plates were either only pretreated or both pre- and post-treated with filtered (0.22 µM) culture supernatants harvested from mid-exponential phase cultures of *S. pneumoniae* (TIGR4) or bacterial cell lysates prepared from the same strain at different dilutions (1∶1, 1∶10, and 1∶100) for 30 min at 37°C. Medium (THY) used to grow pneumococci was included as a control to treat cells at respective dilutions. Cells were washed with PBS and infected with A/swine/Ohio/24366/07 at different MOIs (10, 1, 0.1, and 0.01) in DMEM containing antibiotic with no serum. After 1 hr of viral adsorption the unbound virus was discarded, and the wells designated for post-treatment were again treated with pneumococcal products at the same dilutions. Cell culture supernatants harvested at 8, 16, 24, and 36 hr post-infection were subsequently assayed for viral titers using MDCK cells. Initially, we scored the plate for IAV infection based on virus induced cytopathic effect (CPE) using microscopy [18]. But, as pneumococcal products induced morphological changes in the epithelial cells, especially when used at 1∶1 and 1∶10 dilutions, we used only the IFA method to determine viral titers in all the further experiments. Representative data are shown in Table 1.

### Effect of live *S. pneumoniae* on IAV replication in epithelial cells

Four epithelial cell lines (MDCK, MK1-OSU, A549, and D562) were chosen to investigate the effect of *S. pneumoniae* on replication of six IAV strains. For MDCK, MK1-OSU, and A549 cells, all 12 pneumococcal strains were used at two concentrations (7.5×10^2^ and 7.5×10^4^ CFUs per well) for 1 hr pretreatment. Later, only four pneumococcal strains were randomly selected to analyze in D562 cells using the same experimental design employed for the other three cell lines. THY and DMEM medium were included as controls. After pretreatment the six IAV strains were added to designated wells, at the MOIs selected based on an earlier study (Quantification of IAV and *S. pneumoniae* for pretreatment and infection study). The IFA was performed to enumerate virus infected cell induced FFU at 20 hr post-infection. This experiment was performed three times in triplicate.

### Statistical analysis

All data were expressed as the mean value ± standard error of mean (SEM). Statistical analyses were performed using GraphPad Instat 5.0 Prism software by applying the Welch corrected paired *t*-test to determine the statistical significance (p<0.05).

## Results

### Standardization of a calibration curve to quantify 12 different *S. pneumoniae* strains

**Figure 1:**
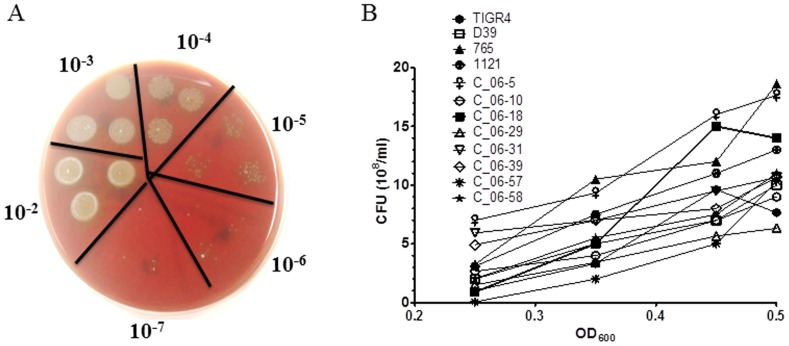
Calibration of a standard curves to quantify different strains of *S. pneumoniae.* Twelve different strains of *S. pneumoniae* were grown in THY medium and CFUs quantified based on OD value over the mid-exponential phase of growth. (A) A representative blood agar plate showing the number of CFUs at 16 hr of incubation in a series of 10-fold diluted *S. pneumoniae* broth culture in triplicate is shown. (B) Calibration curves were plotted for each strain to quantify 12 different *S. pneumoniae* strains in CFUs per mL over the OD_600_ values from 0.2 and 0.6. Each data point on the graph represents the average value of two experiments.

This initial study was performed twice to generate a standard curve for each bacterial strain, which was used subsequently to determine the bacterial CFUs. In this experiment, all 12 *S. pneumoniae* strains were grown successfully by making starter cultures. Serial dilutions were carried out to determine the number of CFUs per mL (Figure 1, *A*). A calibration curve for each *S. pneumoniae* strain was drawn to determine the concentration of the bacteria in CFUs per mL corresponding to an absorbance measurement at OD_600_ (Figure 1, *B*). The value of R^2^ in the calibration equation of each of 12 strains was more than 0.97 (data not shown), indicating the presence of a linear relationship for each strain between OD_600_ (0.25 to 0.5) and CFUs per mL (Figure 1, *B*). The same 12 calibration curves were used to determine the number of bacteria used in the study described below.

### Immunofluorescence microscopy of four epithelial cell lines infected with different IAV strains

**Figure 2:**
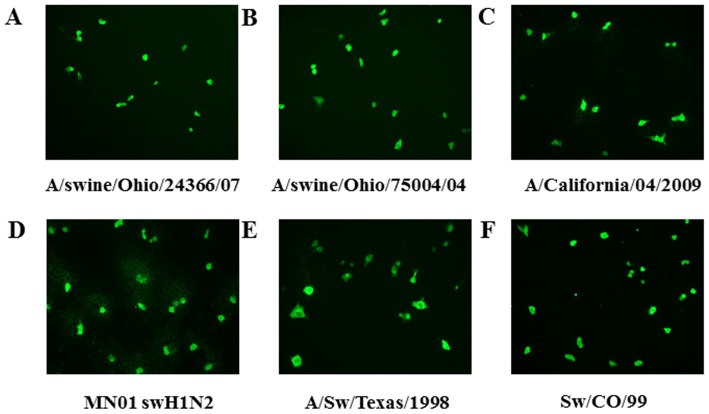
Immunofluorescence pictures of different IAV strains. MDCK cells were infected with indicated IAV strains at an approximate MOI of 0.01. The inoculum of each IAV strain was adjusted to obtain approximately 50–150 fluorescent focal units (FFU) per well following infection for 20 hr. Each FFU is from a single or multiple IAV infected cells in a single fluorescent area.

The MDCK cell line is extensively used for influenza studies and has proved to be a particularly effective model for influenza research [12], [21], [22]. It has been demonstrated that a few swine origin H1N1 influenza viruses are zoonotic and transmit to humans [23]. Therefore, in addition to MDCK cells permissive epithelial cell lines derived from both pig and human origin were also used in this study. All four epithelial cell lines were infected with a range of MOI (1, 0.1, 0.01 and 0.001) to determine the required amount of IAV to induce a countable number of FFU (50- 150 per well). Each of the six IAV at an MOI of approximately 0.01 resulted in 50–150 FFUs per well, and hence this MOI was selected for all experiments. A representative IFA picture of MDCK cells infected with each of the six IAV strains is shown (Figure 2). Our results suggest that the six IAV strains replicated well in each of the four cell types, but the size of the FFU plaques varied depending on the cell type and the strain of IAV (data not shown).

### Effect of treatment of MDCK cells with products of *S. pneumoniae* on IAV replication

To evaluate any impact of pneumococcal products on epithelial cells which alter IAV replication, MDCK cells were pre- and/or post-treated with culture supernatants harvested from mid-exponential phase cultures or bacterial cell lysates prepared from a representative pneumococcal strain (TIGR4). There were two steps in this study, the first step was treatment of the cells with pneumococcal products (0.5–24 hr) followed by IAV infection for 20 hr, and in the second step the harvested supernatants were subjected to virus titration using MDCK cells in 96-well plates. We observed morphological changes in most of the epithelial cells in first step of treatment of pneumococcal products diluted 1∶1 and 1∶10, and in 1∶10 diluted second step titration wells; which was confirmed to be not due to IAV infection by immunostaining the cells for IAV proteins (data not shown). Our results suggested the absence of any significant influence of pneumococcal products (TIGR4) on IAV (A/swine/Ohio/24366/07) replication in MDCK cells (Table 1). Therefore, in our subsequent study we analyzed the impact of pretreatment of live pneumococci on IAV replication using only the IFA method.

### Live *S. pneumoniae* had no effect on IAV replication in epithelial cells

**Figure 3:**
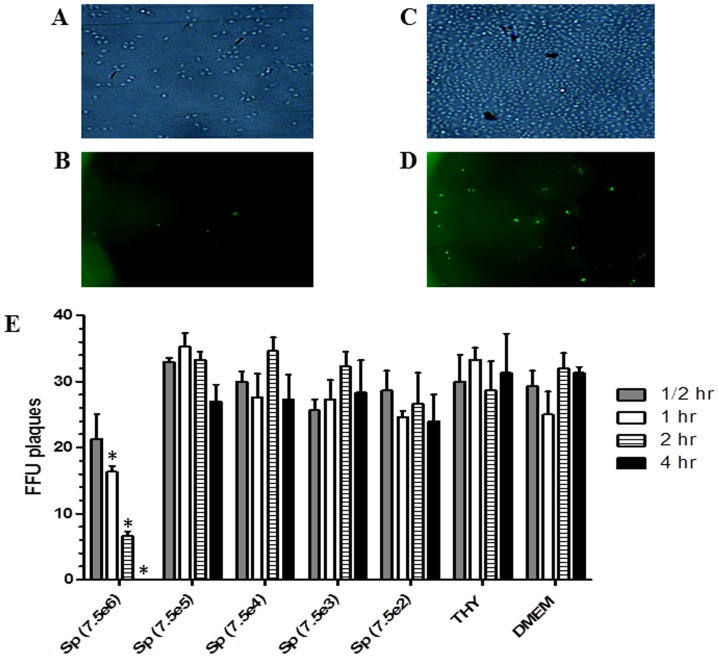
Effect of a range of *S. pneumoniae* inoculums on IAV replication. A representative study with MDCK cells pretreated with live *S. pneumoniae* (TIGR4) for 1 hr followed by infection with an IAV strain (SwH1N1/OH/2007/43266) for 20 hr. Infected cells were analyzed by both light microscopy and immunofluorescence microscopy: (A and C) cells pretreated with 7.5×10^6^ and 7.5×10^5^ CFUs of live TIGR4 strain, respectively, and analyzed by light microscopy; (B and D) similarly treated cells but immunostained and analyzed by immunofluorescence microscopy using a FITC filter. (E) MDCK cells were pretreated with five 10-fold dilutions (7.5×10^6^ to 7.5×10^2^) of live *S. pneumoniae* (TIGR4) for four time points (0.5–4 hr) and the effect on replication of IAV was assessed by IFA. Each bar represents the average FFU in triplicate wells ± SEM. An asterisk indicates a statistically significant (p<0.05) difference between FFU observed following pretreatment with THY medium and *S. pneumoniae* cultures for the stated time period.

As treatment of epithelial cells with pneumococcal products did not alter viral replication, live bacteria were used in subsequent studies. To determine the appropriate bacterial inoculum a titration experiment was performed. Preincubation of MDCK cells with 7.5×10^6^ of *S. pneumoniae* (TIGR4) resulted in gradual cell death in a time-dependent manner (0.5–4 hr) (p<0.01) (Figure 3). We did not perform cell viability assay after the bacterial pretreatment as the cells were still attached in a monolayer. But, when the immunostained plate was observed under the microscope, greater than 80% reduction in the population of adherent cells in wells pretreated with 7.5×10^6^ CFUs of bacteria was observed (Figure 3, *A*). These data suggest the bacteria induced cell toxicity and that the subsequent IAV infection and staining methods detached the sickened cells, leaving very few attached IAV positive FFU (Figure 3, *B*). However, pretreatment of cells with 7.5×10^5^ CFUs of *S. pneumoniae* (TIGR4) had no effect on the cell viability or IAV replication (Figure 3, *C* & *D*). Interestingly, pretreatment of cells with 7.5×10^5^ and lower CFUs of *S. pneumoniae* (TIGR4) did not had any significant effect on the IAV replication (p>0.05) compared to the THY medium control (Figure 3, *E*). The time-dependent reduction in IAV induced FFU plaques in cells pretreated with 7.5×10^6^ CFUs of TIGR4 was due to presence of only a few cells in the wells, and it was significantly less (p<0.01) as compared to both THY and DMEM treated controls (Figure 3, *E*).

**Figure 4:**
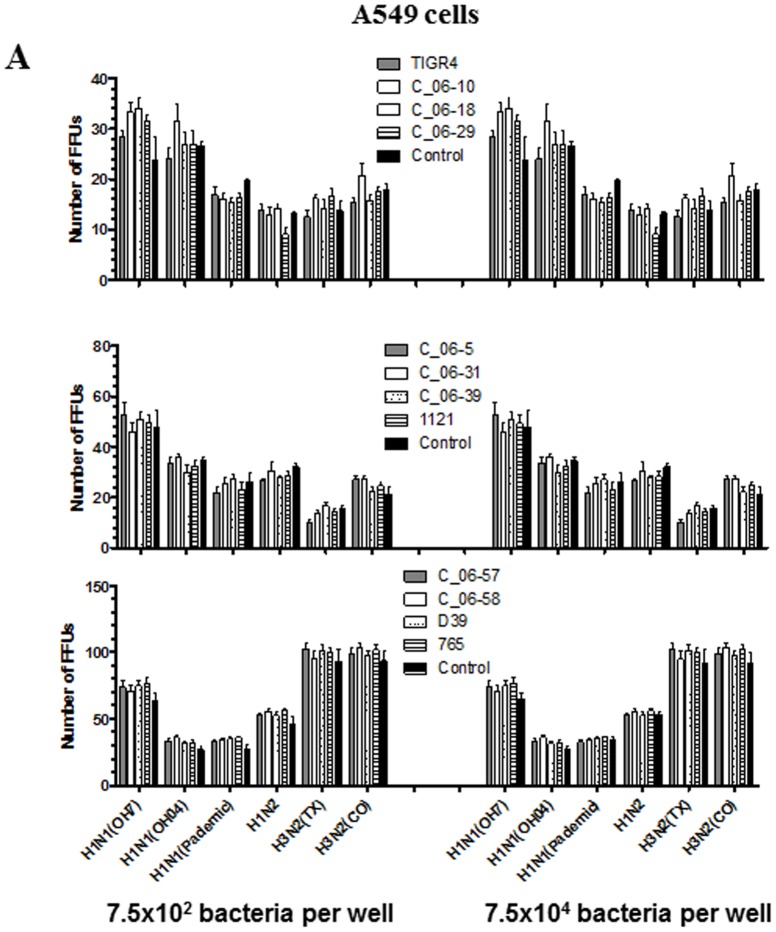
Effect of pretreatment of epithelial cells with 12 live *S. pneumoniae* strains on replication of six IAV strains. The two indicated CFUs of 12 different *S. pneumoniae* strains were used to treat epithelial cell lines: (A) A549; (B) MDCK; (C) MK1; and (D) D562 for 1 hr, and following incubation cells were washed three times with PBS to remove bacteria and then infected with indicated six different IAV strains of both swine and human origin for 20 hr. The virus infected cells were analyzed by the IFA to determine the levels of viral replication. Each bar represents the average number of FFU from two or three independent experiments ± SEM.

Thus, to understand the impact of the 12 different pneumococcal strains (Table 2) on all four selected epithelial cell lines and on IAV replication, we pretreated cells first with 7.5×10^4^ or 7.5×10^2^ CFUs of bacteria per well of a 96-well plate. We verified the integrity of the monolayer of all four cell types after pretreatment with bacteria and IAV infection by microscopy, and found that the cell morphology was comparable to untreated and IAV infected cell monolayers. In the beginning three epithelial cell lines (MDCK, MK1 and A549) were used in the experiment and no significant difference (p>0.05) in the replication of all six IAV strains was detected, with the frequency of FFU plaques comparable to control wells treated with DMEM or THY medium (Figure 4, *A, B* & *C*). Later, selected IAV and pneumococcal strains were used to infect the human pharyngeal carcinoma cell line D562, and the results confirmed that *S. pneumoniae* had no detectable effect on IAV replication in any of the evaluated epithelial cell lines (Figure 4, *D* p>0.05). Statistics and figures shown are from the mean of three independent experiments, performed using 12 pneumococcal strains, six IAV, and four epithelial cell lines (Figure 4).

**Table 2:** Pneumococcal strains used in this study.

Characteristics	Strain	Serotype	Reference
Lys→Thr in RpsL [*rpsL*(*K56T*)] conferring Sm^r^	TIGR4Sm^r^	4	[27]
Laboratory isolate	D39	2	[28]
Clinical isolate	765	6B	[29]
Lys→Thr in RpsL [*rpsL*(*K56T*)] conferring Sm^r^	1121 Sm^r^	23F	[30]
Clinical isolate	C06_05	15A	[31]
Clinical isolate	C06_10	3	[31]
Clinical isolate	C06_18	22F	[32]
Clinical isolate	C06_29	15B	[32]
Clinical isolate	C06_31	23F	[32]
Clinical isolate	C06_39	35F	[32]
Clinical isolate	C06_57	6A/B	[32]
Clinical isolate	C06_58	19A	[32]

## Discussion

Studies have begun to elucidate how viral infections can increase the risk of pneumococcal pneumonia [24], [25]. However, there are no direct studies to determine whether viral titers change following treatment with pneumococci *in vitro*. A study conducted earlier using *S. suis* type 2 was shown to enhance the infection of H3N2 swine IAV on MDCK cells [26]. But, a similar procedure followed with different strains of *S. pneumoniae* failed to enhance replication of six IAV, including swine H3N2. As influenza and pneumococci commonly coinfect the upper respiratory tract of humans we decided to determine whether IAV titers change in the presence of pneumococcal products or with pretreatment of different live pneumococcal strains. For this analysis we made use of a range of IAV strains isolated originally from pigs and humans, belonging to subtypes H1N1, H1N2, and H3N2, including the pandemic 2009 H1N1 virus. As diversity within the pneumococcal population is substantial, the use of a single strain would restrict the conclusions that could be drawn. Therefore, we included 12 different strains of *S. pneumoniae,* eight of which are recent isolates from the human upper respiratory tract (Table 1). Overall, our study represented the interplay of genetically variable IAV and pneumococci routinely found in the human population. Given that we saw no biologically relevant differences in IAV replication with any bacterial and viral combination, it seems likely that the same outcome would be observed with most strains.

We performed our initial studies using treatment of MDCK cells with pneumococcal products and confirmed that the treatment did not have any influence on IAV replication. Data from previous influenza virus pandemics and seasonal influenza outbreaks indicated that coinfections with *S. pneumoniae* and IAV cause increased disease severity. To investigate mechanisms of disease synergy due to these two organisms, several studies have shown that influenza virus induces susceptibility of host cells to *S. pneumoniae* infection. This occurs through induction of secretion of IFN-γ by T cells and reduced secretion of chemokines, associated with activation of NF-κB in alveolar macrophages, mediated through influenza virus [9], [25]. However, until now knowledge on whether *S. pneumoniae* has any role in replication of IAV *in vitro* was unknown.

Pneumococcal-influenza synergism was demonstrated *in vivo* in mice using rodent adapted strains [9], [25]. Influenza infection preceding pneumococcal challenge primed the development of bacterial pneumonia and led to 100% mortality [25]. In a study when infant mice were colonized with *S. pneumoniae* and subsequently infected with IAV three days later, increased pneumococcal colonization and disease in the presence of IAV was noticed, associated with significantly reduced viral titers in nasopharynx compared to control mice [9]. In yet another investigation, mice were infected with IAV followed by *S. pneumoniae*; viral titers initially increased and then declined slowly [10]. Recently, it was demonstrated that *S. pneumoniae* enhances the human metapneumovirus infection in polarized bronchial epithelial cells *in vitro* [11]. However, there is no direct evidence showing the influence of *S. pneumoniae* on the replication of IAV *in vitro* in epithelial cells [5]. Our study using epithelial cell lines revealed the absence of any influence of live pneumococci preexposure on IAV replication, this is in contrast to the published *in vivo* results in rodents [9], [10]. The observed discrepancy appears to be due to the absence of secreted host factors from monolayer cells. Thus, *in vitro* IAV replication in cell lines during coinfections may not be a true representation of the *in vivo* situation.

In this study, pretreatment of MDCK cells with 7.5×10^6^ CFUs of live *S. pneumoniae* resulted in gradual cell death in a time-dependent manner. Pretreatment of cells with 7.5×10^5^ and lower CFUs of *S. pneumoniae* had no detectable effect on health of the cells, and also did not have any noticeable influence on IAV replication. Although, some of the IAV strains replicated better in some cell lines (100–150 FFU) compared to others (20–50 FFU) (Fig. 4), reasons for such a huge variation in counted FFU could be attributed to differences in tropism of virus to different epithelial cell types and the effect of live *S. pneumoniae* pretreatment itself.

We also observed subtle differences in number of IAV induced FFU plaques mediated by pretreatment with a few pneumococcal strains on certain cell types. But, none of the comparisons of the number of FFU plaques, with or without pneumococcal pretreatment were statistically significant. Thus, our *in vitro* exhaustive analysis of IAV and *S. pneumoniae* interaction study suggest that preincubation of a small quantity of *S. pneumoniae* with epithelial cells has no detectable effect on IAV replication. The outcome may be different if there is such a coinfection *in vivo* with increased bacterial loads of different virulent strains of pneumococci or IAV in the upper respiratory tract of humans. It is challenging to perform such studies *in vitro* due to cytotoxic effect of both pneumococcal products and live bacteria on host cells. In addition, it is important to consider the effect of activation of TLR signaling in epithelial cells upon treatment with pneumococcal products versus live bacteria on subsequent IAV replication. Further investigations are required using human polarized bronchial epithelial cells, specific bacterial mutants or very low inoculums for extended periods of time to determine the impact *S. pneumoniae* on IAV replication.
