# Correction: Pretreatment of Epithelial Cells with Live *Streptococcus pneumoniae* Has No Detectable Effect on Influenza A Virus Replication *In Vitro*

**DOI:** 10.1371/journal.pone.0095815

**Published:** 2014-04-15

**Authors:** The PLOS ONE Staff

Figure 4 is incomplete. The complete version with all parts of Figure 4 can be viewed below.

**Figure 4:**
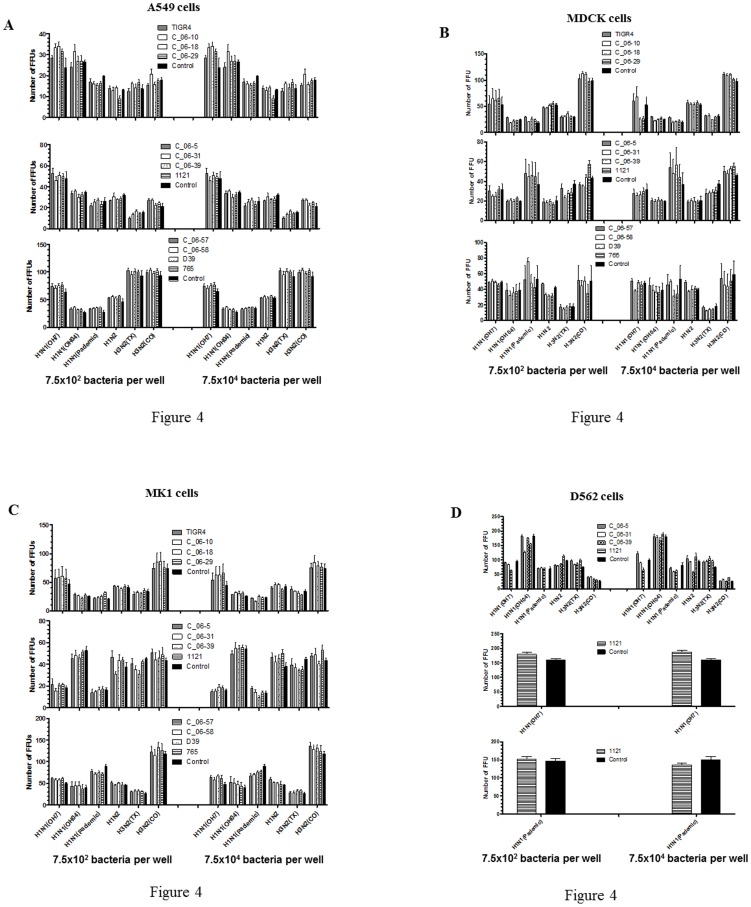
Effect of pretreatment of epithelial cells with 12 live *S. pneumoniae* strains on replication of six IAV strains. The two indicated CFUs of 12 different *S. pneumoniae* strains were used to treat epithelial cell lines: (A) A549; (B) MDCK; (C) MK1; and (D) D562 for 1 hr, and following incubation cells were washed three times with PBS to remove bacteria and then infected with indicated six different IAV strains of both swine and human origin for 20 hr. The virus infected cells were analyzed by the IFA to determine the levels of viral replication. Each bar represents the average number of FFU from two or three independent experiments ± SEM.
